# High-throughput autoantibody analysis in malignant pleural effusion and tuberculosis pleural effusion

**DOI:** 10.1097/MD.0000000000017253

**Published:** 2019-09-20

**Authors:** Fengshuang Yi, Xin Zhang

**Affiliations:** a Medical Research Center; b Department of Respiratory and Critical Care Medicine, Beijing Institute of Respiratory Medicine and Beijing Chao-Yang Hospital, Capital Medical University, Beijing, China.

**Keywords:** autoantibody, high-throughput, malignant pleural effusion, tuberculosis pleural effusion

## Abstract

**Background:**

Malignant pleural effusion (MPE) and tuberculosis pleural effusion (TPE) are 2 kinds of common pleural diseases. Finding efficient and accurate biomarkers to distinguish the 2 is of benefit to basic and clinical research. In the present study, we carried out the first high-throughput autoantibody chip to screen the beneficial biomarker with samples of MPE and TPE and the corresponding serum.

**Methods:**

We collected pleural effusion and serum of patients with MPE (n = 10) and TPE (n = 10) who had been in Beijing Chao-Yang hospital from June 2013 to August 2014. Using RayBio Human Protein Array-G2 to measure the concentration of 487 defined autoantibodies.

**Results:**

Fold changes of Bcl-2-like protein 11 (BIM) autoantibody in MPE-serum/TPE-serum and MPE/TPE groups were 10 (*P* = .019) and 6 (*P* = .001); for decorin autoantibody, MPE-serum/TPE-serum ratio was 0.6 (*P* = .029), and MPE/TPE ratio was 0.3 (*P* < .001).

**Conclusion:**

BIM autoantibody is a promising MPE biomarker by high-throughput autoantibody analysis in MPE and TPE.

## Abstract 2

Supplemental Digital Content is available in the text

## 1 Introduction

Pathogenesis of pleural effusion with exudates needs to be extensively investigated for so many causes will lead to the disease.^[1]^ Pleural malignant disease and tuberculosis pleurisy are 2 challenging conditions to identify when facing with recurrent undiagnosed exudate.^[1–3]^ The annual incidence was about 15,000 cases in the year of 2000 for malignant pleural effusion (MPE) in the United States,^[4]^ and MPE shortened the life expectancy of lung cancer patients, severely,^[5]^ but there is no beneficial fluid biomarker for clinical use for MPE.^[1]^ Although sputum cultures, adenosine deaminase, and inerleukin-27 marker provide benefit for tuberculosis pleural effusion (TPE) diagnosis,^[6–8]^ not all centers afford the available technique. Novel pleural fluid markers should be exploited to provide more effective method for diagnosis of pleural effusion.

Autoantibodies are kinds of antibodies produced by immune system, they react with self and non-self antigens, which participate in variety of activities, and their dysfunction may also lead to many diseases.^[9]^ The autoantibodies have the potential role to indicate the disease status and its clinical evolution prediction.^[10]^ It has been reported that detection of platelet autoantibody provided benefit to guide for optimizing thrombocytopenia treatment.^[11]^ Autoantibodies may be useful blood-based biomarkers for early-stage Parkinson disease diagnosis.^[12]^ Studies on autoantibodies with cancer patients suggested the autoantibodies in biological fluids to be useful diagnosis marker and therapy target.^[9,13]^ In recent years, autoantibodies derived from pleural effusion have been tested for diagnosis of lupus pleuritis and tuberculosis.^[14–18]^ Our present study use high-throughput array method to profile the autoantibodies in MPE, TPE, and also in corresponding serum, which may provide novel biomarker or target for MPE and TPE diagnosis or treatment.

## 2 Patients recruited

The studies is under the proven of ethics committees Beijing Chao-Yang hospital, Capital Medical University, and all participants recruited from June 2013 to August 2014 provided written informed consent. MPE patients were diagnosed by demonstration of malignant cells in pleural fluid and/or on pleural biopsy specimens, and all of these were adenocarcinomas, histologically. TPE patients were diagnosed by Ziehl-Neelsen stains or Lowenstein-Jensen cultures of pleural fluid; sputum or pleural biopsy specimens were positive if granulomas were present in the parietal pleural biopsy specimens. All patients recruited had not received any anticancer treatment, antituberculosis therapy, corticosteroids, or other nonsteroidal anti-inflammatory drugs when collecting samples.

## 3 Sample collection and processing

Pleural fluid and blood were collected before receiving any treatment and were centrifuged at 1500 rpm at 4°C for 10 minutes, and the cell-free supernatant was analyzed immediately or stored at −80°C waiting for future measurement.

## 4 Detection of autoantibodies by microarray

For RayBio Human Protein Array-G2, 487 native or recombinant proteins are spotted onto the surface of a solid glass slide support, which can monitor the presence of autoantibodies. We carried out the profiling test following RayBio Human Protein Array G Series protocol of detecting autoantibodies, and capture the signals using laser scanner, Axon GenePix by cy3 channel.

## 5 Data extraction and normalization

The captured array signal was extracted with GenePix. The signal intensities can simply be analyzed by importing the fluorescence values into RayBio analysis tool (Cat. # 02-PAH-G2). Then the data obtained from the array image will be automatically computed. The positive control is a controlled amount of biotinylated protein printed on the arrays in triplicate; the raw data normalization is used to compare data between different samples by accounting for the differences in signal intensities of the positive control spots on those arrays. The normalized values are calculated as follows: 
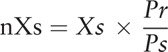

- Pr: the average signal density of the positive control spots on the reference array (r).
- Ps: the average signal density of the positive control spots on the sample array (s).
- Xs: the signal density for a particular spot (X) on sample array (s).
- nXs: the normalized Xs value.

## 6 Statistical analysis

Data were expressed as mean ± SD, changes of different autoantibodies in different groups were calculated by the concentration ratio between different groups. Comparisons of data between different groups were performed using Mann-Whitney *U* test. All statistical analyses were performed by SPSS 20.0 (SPPSS Inc, Chicago, IL); a *P* value <.05 was considered as statistically significant.

## 7 Results

In this study, PE and blood from 10 MPE and 10 TPE patients were collected to carry out the following analysis. The basic demographic, biochemical, and cytological characteristics in pleural effusions are listed in the Table 1. The expression level of autoantibodies was detected (Supplementary Figure 1 and 2), calculated, and normalized. Concentration ratios between different groups have been calculated and autoantibodies with a *P* value <.05 were listed in Tables 2 and 3.

**Table 1:**
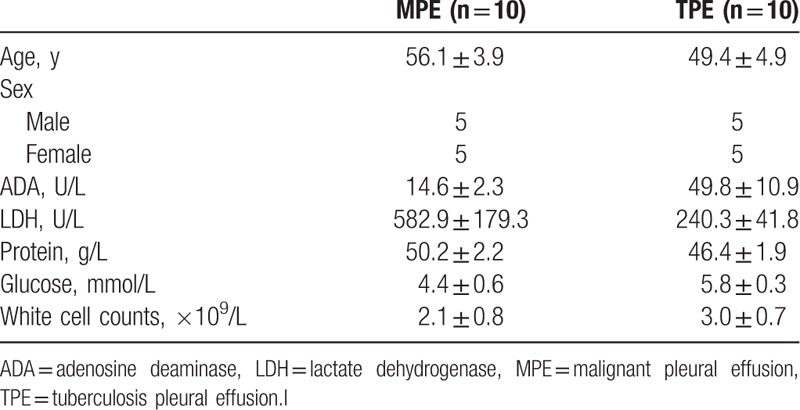
Patient demographics and biochemical and cytological characteristics of pleural effusions.

**Table 2:**
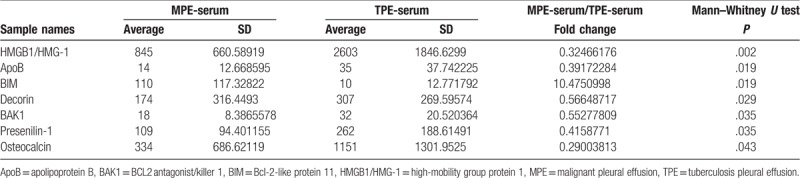
Comparison between MPE-serum and TPE-serum autoantibodies concentration.

**Table 3:**
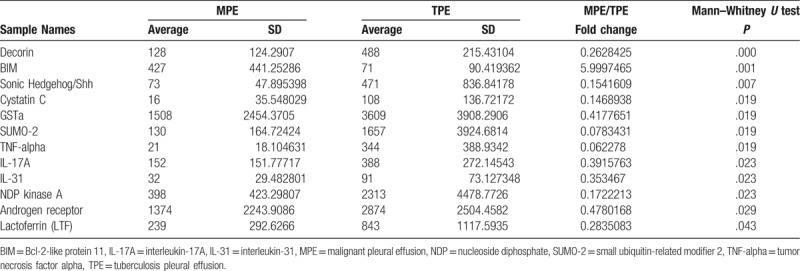
Comparison between MPE and TPE autoantibody concentrations.

**Figure 1:**
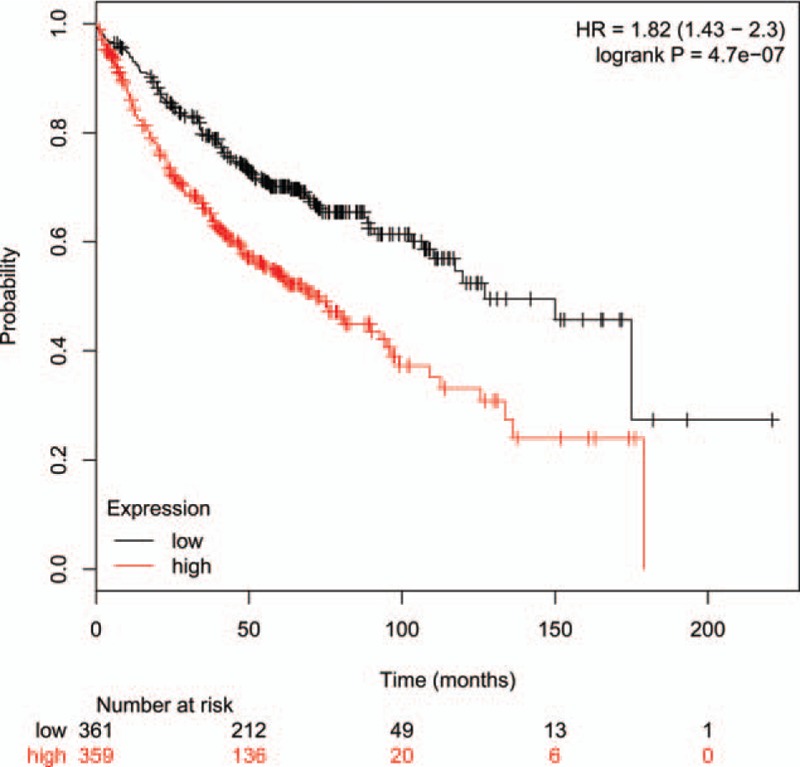
Kaplan–Meier plots showing overall survival (OS) of lung adenocarcinomas patients stratified by high or low Bcl-2-like protein 11 (BIM) expression. Kaplan–Meier survival curves showing OS of lung cancer patients with BIM expression levels. Data are from 720 patients for which survival information was available. *P* = 4.7e-07, log-rank test. HR = hazard ratio.

**Figure 2:**
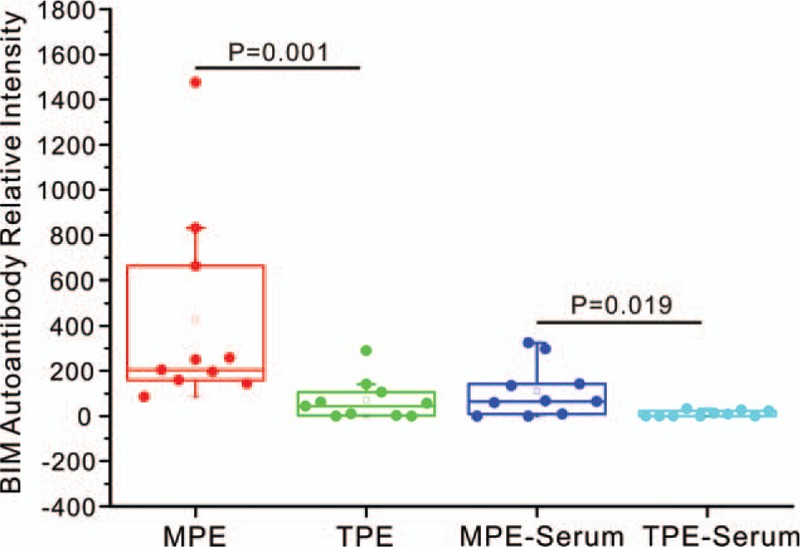
Whisker/box plot showing distribution of BIM autoantibody intensity from different samples. To each group, n = 10. MPE/ TPE, *P* = .001; MPE-serum/TPE-serum, *P* = .019, Mann–Whitney *U* test. BIM = Bcl-2-like protein 11, MPE = malignant pleural effusion, TPE = tuberculosis pleural effusion.

Analysis between MPE-serum/TPE-serum ratio and MPE/TPE ratio showed that most of the MPE-serum/TPE-serum and MPE/TPE fold change values were <1, which suggested lower autoantibodies concentration in MPE-serum and MPE compared with TPE-serum and TPE. What was interesting, fold changes of Bcl-2-like protein 11 (BIM) autoantibody in MPE-serum/TPE-serum and MPE/TPE groups were 10 (*P* = .019, Table 2) and 6 (*P* = .001, Table 3), respectively. Whisker/box plot was drawn to show the BIM autoantibody concentration distribution among different groups (Fig. 2). The result indicated that BIM autoantibody concentration was higher both in MPE and MPE-serum than that in TPE and TPE-serum and BIM may function in the pathogenesis of MPE. Analysis of the published lung cancer datasets^[19]^ showed that lung adenocarcinomas patients with higher levels of BIM expression had significantly shorter overall survival (OS) than did those expressing lower levels of BIM (Fig. 1). These results support the notion that BIM functions as a tumor-promoting factor in lung cancer progression.

Decorin was another interesting autoantibody detected in both groups, although its concentration was lower in MPE and MPE-serum compared to TPE and TPE-serum; MPE-serum/TPE-serum ratio was 0.6 (*P* = .029, Table 2), and MPE/TPE ratio was 0.3 (*P* < .001, Table 3). As Decorin autoantibody concentration was higher both in TPE and TPE-serum when compared with MPE and MPE-serum, respectively, it may function in the pathogenesis of TPE and be a promising biomarker for TPE.

## 8 Discussion

It is challenging to distinguish between malignant and TPE, in the present study; we carried out the first high-throughput autoantibody screening by using RayBio Human Protein Array-G2 chip with 487 defined autoantibodies. In the analysis of the results, most listed autoantibodies concentration was lower in MPE-serum and MPE compared with TPE-serum and TPE, which may indicated the immune status was more active in TPE patients than that in MPE patients. We found 2 interesting autoantibodies, BIM and Decorin. Both in MPE-serum/TPE-serum and MPE/TPE groups, BIM autoantibody was the only target with the fold-change value >1, which suggested that its concentration in MPE is higher than that in TPE, and the results are similar when compared between MPE-serum and TPE-serum. Decorin autoantibody was the other target listed in both groups; its concentration is higher in TPE-serum and TPE than that in MPE-serum and MPE.

BIM, also called Bcl-2-like protein 11, is a protein that in humans is encoded by the *BCL2L11* gene.^[20]^ BIM functions in a variety of physiology and pathology events, starvation or drug could induce dual role of BIM in apoptosis and autophage, BIM is involved in autoimmune diseases, neurogenerative disorders, diabetes, fibrosis, cancer, and myelo/lymphoproliferative disorders.^[21]^ Up to now, there are several kinds of compounds designed based on BIM-BH3 domain to be anti-cancer agent. ABT-737,^[22,23]^ ABT-263,^[24]^ and A-1210477^[25]^ either are the preclinical or clinical anti-cancer drugs, which are efficient against tumors. Although GX15-070 alone is not efficient as a single agent to treat cancer, but it can increase the susceptibility of multiple myeloma cells to other chemotherapeutic drugs.^[26]^ Whisker/box plot analysis was done to visualize the effects of potential outliers on the conclusion, and the conclusion was the same when omitting the one of the possible outlier in MPE. BIM autoantibody is the only candidate which concentration are both higher in MPE-Serum and MPE when compared with TPE-Serum and TPE by using high-throughput autoantibody chip screening. The phenomenon suggested that BIM autoantibody may function in the pathogenesis of MPE, and be a promising biomarker for MPE. Decorin is a prototype small leucine-rich proteoglycan; it functions in collagen angiostasis, fibrillogenesis, wound repair, tumor growth, autophagy, and inflammation.^[27]^ In this study, Decorin is the only autoantibody whose concentration is higher in both TPE-Serum and TPE compared with MPE-serum and MPE, respectively, and it may play a role in the pathogenesis of TPE.

As the present study was an exploratory and tentative research, the samples of pleural effusion and blood were obtained from only 10 MPE and 10 TPE patients. More patients should be recruited in the study and more functional test of BIM and Decorin on MPE and TPE should be done in the future.

## Author contributions

**Formal analysis:** Fengshuang Yi, Xin Zhang.

**Investigation:** Fengshuang Yi, Xin Zhang.

**Methodology:** Xin Zhang.

**Project administration:** Fengshuang Yi.

**Resources:** Fengshuang Yi.

**Software:** Xin Zhang.

**Supervision:** Fengshuang Yi.

**Validation:** Xin Zhang.

**Writing – original draft:** Fengshuang Yi, Xin Zhang.

**Writing – review & editing:** Fengshuang Yi, Xin Zhang.

## Supplementary Material

Supplemental Digital Content
