# Supplementary material for: High-throughput autoantibody analysis in malignant pleural effusion and tuberculosis pleural effusion
Source: Medicine (Baltimore). 2019 Sep 20;98(38):e17253. doi: 10.1097/MD.0000000000017253 (PMC6756716; doi:10.1097/MD.0000000000017253)
Supplement: Supplemental Digital Content [file medi-98-e17253-s001.doc]

**Supplementary Figure 1: The malignant pleural effusion and tuberculosis pleural effusion fluorescence signal intensities spots on arrays.**

**
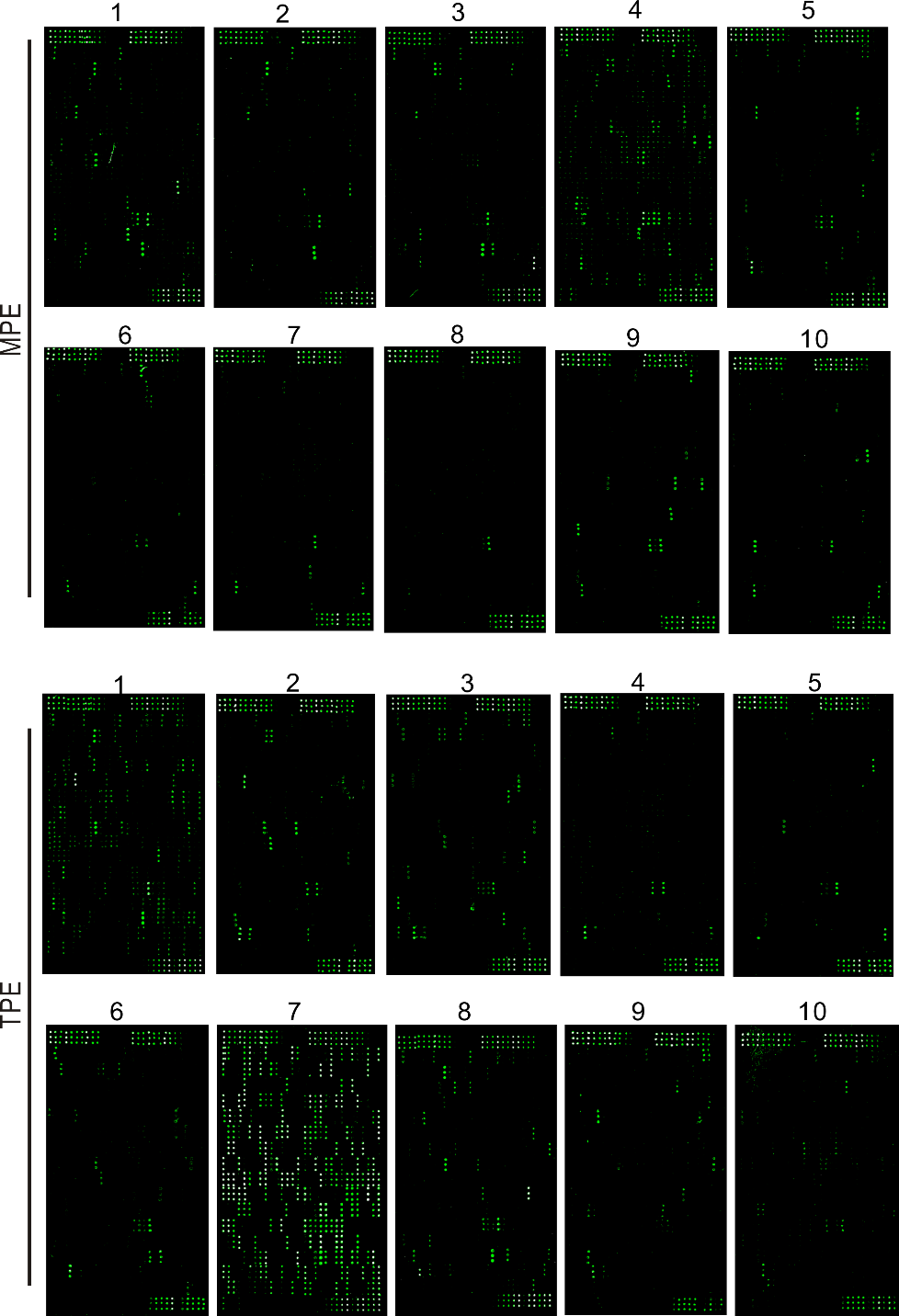
**

**Supplementary Figure 2: The fluorescence signal intensities spots of serum from malignant pleural effusion and tuberculosis pleural effusion patients and healthy control on arrays.**

**
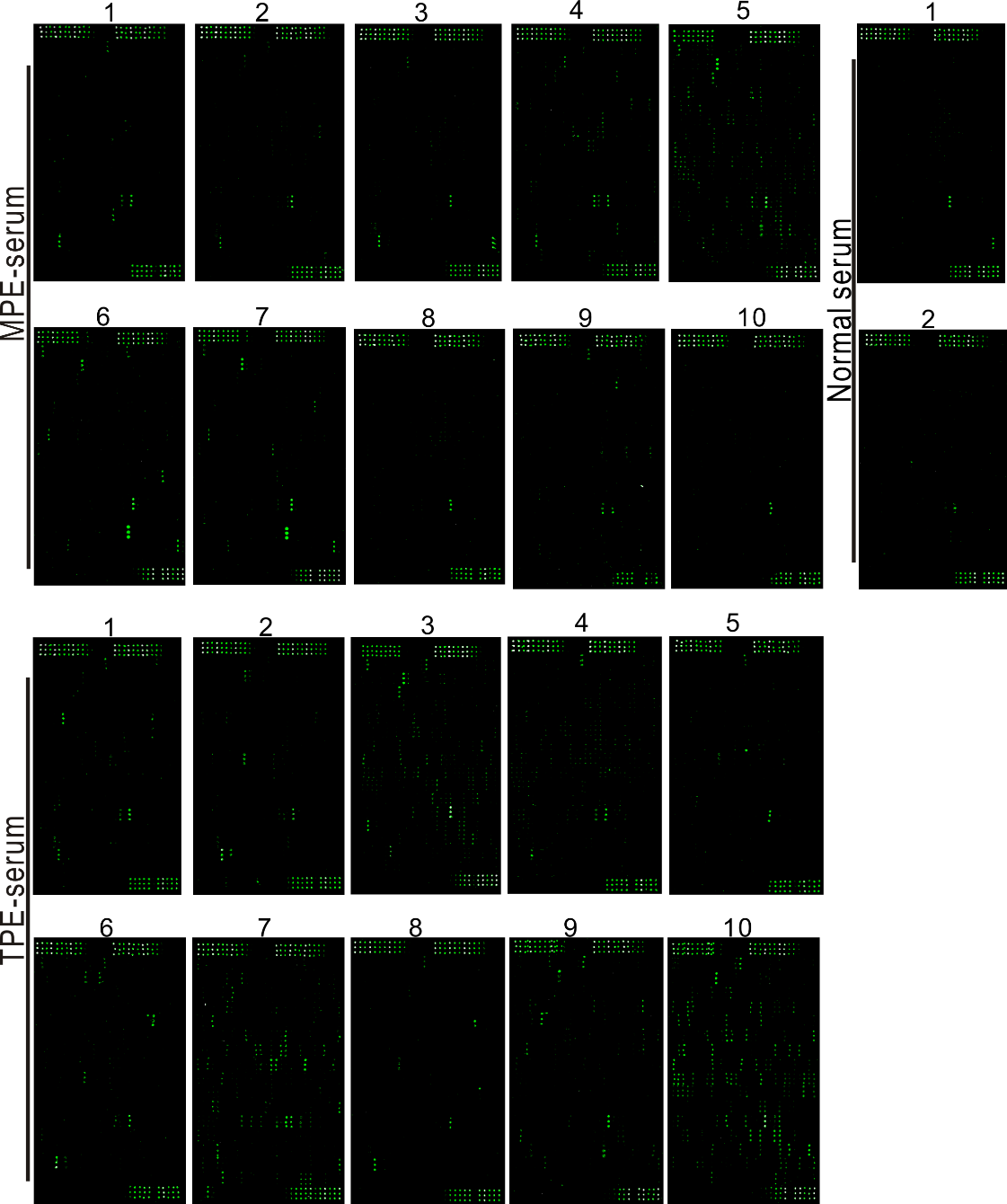
**
